# Supplementary material for: Marker-Assisted Selection for Early Maturing E Loci in Soybean Yielded Prospective Breeding Lines for High Latitudes of Northern Kazakhstan
Source: Biomolecules. 2023 Jul 18;13(7):1146. doi: 10.3390/biom13071146 (PMC10377072; doi:10.3390/biom13071146)
Supplement: Supplementary file 1 [file biomolecules-13-01146-s001.zip › RY-Supplementary-material-S1-S5-submitted-18-07-2023.pdf]

Supplementary Materials to the paper

Yerzhebayeva, R.; Didorenko, S.; Amangeldiyeva, A.; Daniyarova, A.; Mazkirat, S.; Zinchenko, A.; Shavrukov, Y. Marker-Assisted Selection for Early Maturing *E* Loci in Soybean Yielded Prospective Breeding Lines for High Latitudes of Northern Kazakhstan. *Biomolecules* **2023**.

Supplementary Material S1. Sequences of primers for the used molecular markers and PCR conditions for allele identification in four E genes controlling sensitivity to photoperiod in soybean plants.

Gene	Molecular markers		
	**Name**	**Primer nucleotide sequences**	**PCR conditions**
E1	Satt557	F: GCGGGATCCACCATGTAATATGTG<br>R: GCGCACTAACCCTTTATTGAA	95 C – 5 min; 35 cycles (92 C – 30 sec, 58°C – 30 sec, 72°C – 45 sec); 72°C – 7 min
	Satt365	F: TGCTCCCCTCTGCCTTTTTTTCTATTTT<br>R: AAGGATGAGTTTGATAAACATGAATGAAGAA	
E3	Satt 229	F: TGGCAGCACACCTGCTAAGGGAATAAA<br>R: GCGAGGTGGTCTAAAATTATTACCTAT	95 C – 5 min; 35 cycles (94 C – 30 sec, 58°C – 45 sec, 72°C – 45 sec); 72°C – 7 min
E4	PhyA2	F: AGACGTAGTGCTAGGGCTAT<br>R1: GCATCTCGCATCACCAGATCA<br>R2: GCTCATCCCTTCGAATTCAG	94 C – 5 min; 30 cycles (94 C – 20 sec, 58°C – 20 sec, 72°C – 1 min); 72°C – 5 min
E7	Satt 100	F: ACCTCATTTTGGCATAAA<br>R: TTGGAAAACAAGTAATAATAACA	95 C – 5 min; 35 cycles (92 C – 45 sec, 53°C – 1 min, 72°C – 1 min 20 sec); 72°C – 7 min
	Satt 319	F: CAACTCAGTAGGGGTCAATAACAA<br>R: TGAAATAGGGAAAATAAGGGAACA	95 C – 5 min; 35 cycles (92 C – 25 sec, 53°C – 25 sec, 72°C – 35 sec); 72°C – 10 min

Supplementary material S2. Results and efficiency of hybridization.

N	Hybrid	Number of flowers used for hybridization	Number of hybrid pods	Number of hybrid seeds	Hybridization efficiency (%)	Number of F_1 hybrid plants with purple flower	Number of true hybrid plants
1	Birlik KB × Rana	30	7	10	23.3	9	3
2	Birlik KB × Toury	30	7	10	23.3	9	4
3	Birlik KB × Pripyat	30	5	7	16.7	7	1
4	Birlik KB × Grignon 5	30	7	10	23.3	9	4
5	Birlik KB × Pamyat YGK	30	7	9	23.3	9	7
6	Birlik KB × Soer 345	30	5	7	16.7	7	2
7	Birlik KB × Altom	30	0	0	0.0	0	0
8	Zara ×Bara	30	4	7	13.3	7	3
9	Zara ×Maleta	30	4	7	13.3	6	5
10	Zara ×Soer 3	30	6	6	20.0	5	4
11	Zara ×Ustya	30	2	3	6.7	3	2
12	Zara ×Yaselda	30	2	2	6.7	2	1
13	Zara ×Maple Amber	30	0	0	0.0	0	0
14	Zara ×Fiskeby III	30	0	0	0.0	0	0
15	Zara ×Jhony	30	3	5	10.0	4	2
	**Total**	**450**	**59**	**83**	**13.1**	**77**	**38**

Supplementary material S3. Growth and yield traits in plants from hybrid populations grown in southern Kazakhstan.

N	Type	Name	Plant height (cm)	First pod height (cm)	Pod number per plant	1000 seeds weigh (g)	Seed weight per plant (g)	Time to maturity (days)
1	Isoline	Harosoy OT94-47	32,8±3,3	7,2±2,3	21,2±6,2	168,0±4,9	5,4±0,1	96
2	Isoline	Harosoy OT89-5	55,4±3,5	8,0±0,6	31,2±6,8	180,8±2,6	7,5±1,7	99
3	Isoline	cv. Harosoy	58,2±4,4	5,2±1,1	54,2±15,6	186,2±3,3	20,6±4,1	118
4	♀	Birlik KV	39,1±6,4	2,8±1,4	36,3±13,1	170,0±21,5	11,8±4,6	112
5	HP*	Birlik KV x Rana	35,3±9,8	4,4±1,7	40,2±25,1	168,9±18	12,4±7,9	107
6	♂	Rana	28,0±3,9	7,8±4,7	14,0±4,0	152,7±20,9	5,8±0,6	104
7	HP	Бірлік КВ x Pripyat	30,2±7,1	5,5±2,1	19,1±12,6	189,9±23,3	7,4±5,1	108
8	♂	Pripyat	34,6±3,9	4,3±2,7	21,5±5,4	178,3±23,4	7,8±2,0	116
9	HP	Birlik KV x Gignon 5	29,6±10,5	5,0±2,3	20,9±19,0	183,6±44,3	8,5±8,2	108
10	♂	Gignon 5	30,5±5,4	4,5±2,4	22,4±11,0	185,47,5	7,3±3,7	128
11	HP	Birlik KV x Pamyat YGK	53,5±10,5	7,7±2,8	28,321,3	192,6±26,7	11,3±7,7	126
12	♂	Pamyat YGK	55,6±8,1	7,6±3,0	44,5±22,3	186,3±15,2	19,8±10,2	129
13	HP	Birlik KV x Toury	33,9±10,1	6,2±2,5	21,4±16,9	180,5±32,8	7,4±5,9	107
14	♂	Toury	41,8±10,9	8,4±1,1	28,7±18,6	204,3±15,7	9,9±6,9	110
15	HP	Birlik KV x Soer 345	32,4±10,2	5,7±2,6	25,2±17,2	163,9±3,2	8,5±6,4	113
16	♂	Soer 345	30,8±8,0	5,5±2,5	19,7±8,8	164,7±23,9	7,3±4,1	116
17	♀	Zara	63,6±8,6	5,3±1,6	48,2±13,7	167,0±23,4	16,5±5,4	116
18	HP	Zara x Bara	45,1±7,5	7,3±1,3	33,0±9,8	206,1±18,3	9,8±1,9	112
19	♂	Bara×	25,1±4,1	9,3±1,2	10,0±2,9	210,1±20,2	6,8±1,7	110
20	HP	Zara x Maleta	57,3±16,2	5,8±2,8	37,0±14,9	166,3±27,9	13,1±6,1	116
21	♂	Maleta	38,0±7,3	5,0±0,6	29,6±4,9	154,2±13,6	9,0±0,9	85
22	HP	Zara x Ustya	52,1±10,9	4,7±2,3	48,9±20,6	158,5±23,9	17,2±7,9	119
23	♂	Ustya	27,9±3,6	5,1±1,2	21,1±6,5	180,7±14,7	7,5±2,6	110
24	HP	Zara x Soer 3	47,9±7,7	6,7±3,7	40,8±18,3	158,6±21,1	13,8±6,6	121
25	♂	Soer 3	25,8±1,5	4,5±0,5	12,8±0,8	161,3±6,5	13,3±10,2	94
26	HP	Zara x Jhony	68,1±13,1	6,1±1,2	65,7±21,0	173,4±15,5	24,1±8,7	114
27	♂	Jhony	38,9±11,9	5,1±1,4	23,7±11,5	192,7±11,3	8,1±4,4	110

Note: HP*, Hybrid population

Supplementary material S4. The list of the 103 identified soybean hybrid breeding lines, selected and studied for allelic polymorphism in *E* genes, flowering and maturation in northern Kazakhstan, Kostanay, 2022.

Note: Time to maturity over 121 days is indicated in Bold for genotypes that did not complete their maturation during growth period.

N	Hybrid	Breeding line ID	Alleles of *E* genes				Genotype	Flowering time (days)	Time to maturity (days)
			E1	***E3***	***E4***	***E7***			
1	Birlik KV × Toury	P-6/3-2	*e1*	*E3*	*E4*	*e7*	*e1 E3 E4 e7*	33	106
2	Birlik KV × Pripyat	P-2/7-1	*e1*	*E3*	*E4*	*e7*	*e1 E3 E4 e7*	35	114
3	Birlik KV × Pripyat	P-2/5-4	*e1*	*E3*	*e4*	*e7*	*e1 E3 e4 e7*	36	92
4	Birlik KV × Rana	P-1/11-5	*e1*	*e3*	*E4*	*e7*	*e1 e3 E4 e7*	37	94
5	Birlik KV × Rana	P-1/2-3	*e1*	*e3*	*E4*	*e7*	*e1 e3 E4 e7*	37	94
6	Birlik KV × Rana	P-1/10-2	*e1*	*e3*	*E4*	*e7*	*e1 e3 E4 e7*	38	94
7	Birlik KV × Rana	P-1/2-2	*e1*	*e3*	*E4*	*e7*	*e1 e3 E4 e7*	38	94
8	Birlik KV × Rana	P-1/10-5	*e1*	*e3*	*E4*	*e7*	*e1 e3 E4 e7*	38	95
9	Birlik KV × Rana	P-1/8-4	*e1*	*e3*	*E4*	*e7*	*e1 e3 E4 e7*	38	96
10	Birlik KV × Rana	P-1/10-3	*e1*	*e3*	*E4*	*e7*	*e1 e3 E4 e7*	38	96
11	Birlik KV × Rana	P-1/7-3	*e1*	*e3*	*E4*	*e7*	*e1 e3 E4 e7*	38	**130**
12	Zara × Jhony	P-13/1-1	*E1*	*E3*	*e4*	*E7*	*E1 E3 e4 E7*	38	**134**
13	Zara × Maleta	P-19/1-2	*E1*	*E3*	*E4*	*E7*	*E1 E3 E4 E7*	38	113
14	Birlik KV × Gignon 5	P-3/3-1	*e1*	*E3*	*E4*	*e7*	*e1 E3 E4 e7*	39	102
15	Birlik KV × Gignon 5	P-3/1-2	*e1*	*E3*	*E4*	*e7*	*e1 E3 E4 e7*	39	106
16	Birlik KV × Gignon 5	P-3/2-1	*E1*	*E3*	*E4*	*E7*	*E1 E3 E4 E7*	39	109
17	Birlik KV × Pamyat YGK	P-10/4-2	*e1*	*E3*	*E4*	*e7*	*e1 E3 E4 e7*	39	**131**
18	Birlik KV × Rana	P-1/4-1	*e1*	*E3*	*E4*	*e7*	*e1 E3 E4 e7*	39	103
19	Birlik KV × Rana	P-1/3-4	*e1*	*E3*	*E4*	*e7*	*e1 E3 E4 e7*	39	103
20	Birlik KV × Rana	P-1/2-1	*e1*	*e3*	*E4*	*e7*	*e1 e3 E4 e7*	39	**140**
21	Birlik KV × Toury	P-6/4-1	*e1*	*E3*	*E4*	*e7*	*e1 E3 E4 e7*	39	94
22	Zara × Maleta	O-47/31	*e1*	*E3*	*E4*	*e7*	*e1 E3 E4 e7*	39	103

23	Zara × Maleta	P-19/6-5	*e1*	*E3*	*e4*	*e7*	*e1 E3 e4 e7*	39	116
24	Birlik KV × Gignon 5	P-3/2-4	*e1*	*E3*	*E4*	*e7*	*e1 E3 E4 e7*	40	102
25	Birlik KV × Gignon 5	P-3/7-4	*e1*	*E3*	*E4*	*e7*	*e1 E3 E4 e7*	40	116
26	Birlik KV × Gignon 5	P-3/4-6	*e1*	*E3*	*E4*	*e7*	*e1 E3 E4 e7*	40	117
27	Birlik KV × Pripyat	P-2/6-1	*e1*	*E3*	*E4*	*e7*	*e1 E3 E4 e7*	40	103
28	Birlik KV × Pripyat	P-2/10-5	*e1*	*E3*	*E4*	*e7*	*e1 E3 E4 e7*	40	117
29	Birlik KV × Rana	P-1/6-4	*E1*	*e3*	*E4*	*E7*	*E1 e3 E4 E7*	40	101
30	Birlik KV × Rana	P-1/8-1	*e1*	*e3*	*E4*	*e7*	*e1 e3 E4 e7*	40	111
31	Birlik KV × Rana	P-1/3-2	*e1*	*E3*	*E4*	*e7*	*e1 E3 E4 e7*	40	111
32	Birlik KV × Soer 345	P-9/3-6	*E1*	*E3*	*E4*	*E7*	*E1 E3 E4 E7*	40	93
33	Birlik KV × Toury	P-6/2-6	*e1*	*E3*	*E4*	*e7*	*e1 E3 E4 e7*	40	109
34	Birlik KV × Toury	P-6/8-6	*e1*	*E3*	*E4*	*e7*	*e1 E3 E4 e7*	40	110
35	Birlik KV × Toury	P-6/7-9	*e1*	*E3*	*E4*	*e7*	*e1 E3 E4 e7*	40	**135**
36	Zara × Maleta	O-47/32	*e1*	*E3*	*e4*	*e7*	*e1 E3 e4 e7*	40	104
37	Zara × Maleta	P-19/10-1	*e1*	*E3*	*e4*	*e7*	*e1 E3 e4 e7*	40	**135**
38	Zara × Maleta	O-47/41	*e1*	*E3*	*e4*	*e7*	*e1 E3 e4 e7*	40	**130**
39	Zara × Ustya	P-20/2-2	*E1*	*E3*	*E4*	*E7*	*E1 E3 E4 E7*	40	**138**
40	Birlik KV × Pripyat	P-2/8-5	*e1*	*E3*	*e4*	*e7*	*e1 E3 e4 e7*	41	114
41	Birlik KV × Rana	P-1/8-3	*e1*	*e3*	*E4*	*e7*	*e1 e3 E4 e7*	41	101
42	Birlik KV × Rana	P-1/3-1	*e1*	*e3*	*E4*	*e7*	*e1 e3 E4 e7*	41	102
43	Birlik KV × Rana	P-1/9-5	*E1*	*e3*	*E4*	*E7*	*E1 e3 E4 E7*	41	102
44	Birlik KV × Rana	P-1/8-2	*e1*	*e3*	*E4*	*e7*	*e1 e3 E4 e7*	41	103
45	Birlik KV × Rana	P-1/12-1	*e1*	*e3*	*E4*	*e7*	*e1 e3 E4 e7*	41	104
46	Birlik KV × Rana	P-1/7-1	*E1*	*e3*	*E4*	*E7*	*E1 e3 E4 E7*	41	104
47	Birlik KV × Rana	P-1/13-4	*E1*	*E3*	*E4*	*E7*	*E1 E3 E4 E7*	41	106
48	Birlik KV × Rana	P-1/8-1	*e1*	*e3*	*E4*	*e7*	*e1 e3 E4 e7*	41	111
49	Birlik KV × Soer 345	P-9/2-2	*e1*	*E3*	*E4*	*e7*	*e1 E3 E4 e7*	41	**136**
50	Zara × Bara	P-17/2-4	*E1*	*E3*	*E4*	*E7*	*E1 E3 E4 E7*	41	**133**

51	Birlik KV × Rana	P-1/11-1	*e1*	*e3*	*E4*	*e7*	*e1 e3 E4 e7*	42	102
52	Birlik KV × Rana	P-1/7-4	*E1*	*E3*	*E4*	*E7*	*E1 E3 E4 E7*	42	102
53	Birlik KV × Rana	P-1/3-3	*e1*	*e3*	*E4*	*e7*	*e1 e3 E4 e7*	42	102
54	Birlik KV × Rana	P-1/7-5	*E1*	*e3*	*E4*	*E7*	*E1 e3 E4 E7*	42	107
55	Zara × Bara	P-17/3-3	*E1*	*E3*	*E4*	*E7*	*E1 E3 E4 E7*	42	**133**
56	Zara × Maleta	O-47/12	*e1*	*E3*	*e4*	*e7*	*e1 E3 e4 e7*	42	109
57	Zara × Maleta	O-47/42	*E1*	*e3*	*E4*	*E7*	*E1 e3 E4 E7*	42	**138**
58	Zara × Ustya	P-20/5-5	*E1*	*E3*	*E4*	*E7*	*E1 E3 E4 E7*	42	121
59	Birlik KV × Pripyat	P-2/8-9	*e1*	*E3*	*E4*	*e7*	*e1 E3 E4 e7*	43	113
60	Birlik KV × Pripyat	P-2/4-8	*e1*	*E3*	*e4*	*e7*	*e1 E3 e4 e7*	43	114
61	Birlik KV × Rana	P-1/5-1	*E1*	*e3*	*E4*	*E7*	*E1 e3 E4 E7*	43	107
62	Birlik KV × Rana	P-1/5-2	*E1*	*e3*	*E4*	*E7*	*E1 e3 E4 E7*	43	110
63	Birlik KV × Rana	P-1/9-1	*E1*	*e3*	*E4*	*E7*	*E1 e3 E4 E7*	43	112
64	Birlik KV × Rana	P-1/1-4	*E1*	*E3*	*E4*	*E7*	*E1 E3 E4 E7*	43	113
65	Birlik KV × Rana	P-1/9-2	*E1*	*e3*	*E4*	*E7*	*E1 e3 E4 E7*	43	113
66	Birlik KV × Soer 345	P-9/4-8	*E1*	*E3*	*E4*	*E7*	*E1 E3 E4 E7*	43	**130**
67	Zara × Bara	P-17/2-3	*E1*	*E3*	*E4*	*E7*	*E1 E3 E4 E7*	43	**130**
68	Zara × Maleta	O-47/21	*E1*	*E3*	*E4*	*E7*	*E1 E3 E4 E7*	43	114
69	Zara × Ustya	P-20/2-3	*E1*	*E3*	*E4*	*E7*	*E1 E3 E4 E7*	43	121
70	Birlik KV × Rana	P-1/13-5	*E1*	*e3*	*E4*	*E7*	*E1 e3 E4 E7*	45	**130**
71	Birlik KV × Toury	P-6/7-7	*e1*	*E3*	*E4*	*e7*	*e1 E3 E4 e7*	45	**138**
72	Birlik KV × Gignon 5	P-3/2-8	*e1*	*E3*	*E4*	*e7*	*e1 E3 E4 e7*	46	**130**
73	Birlik KV × Pamyat YGK	P-10/1-2	*E1*	*E3*	*E4*	*E7*	*E1 E3 E4 E7*	46	**130**
74	Birlik KV × Rana	P-1/1-3	*E1*	*E3*	*E4*	*E7*	*E1 E3 E4 E7*	46	113
75	Birlik KV × Rana	P-1/1-1	*E1*	*E3*	*E4*	*E7*	*E1 E3 E4 E7*	46	114
76	Birlik KV × Toury	P-6/4-3	*e1*	*E3*	*E4*	*e7*	*e1 E3 E4 e7*	46	**138**
77	Birlik KV × Rana	P-1/5-3	*E1*	*e3*	*E4*	*E7*	*E1 e3 E4 E7*	47	**132**
78	Birlik KV × Soer 345	P-9/6-3	*E1*	*E3*	*E4*	*E7*	*E1 E3 E4 E7*	47	**130**

79	Birlik KV × Pamyat YGK	P-10/4-1	*e1*	*E3*	*E4*	*e7*	*e1 E3 E4 e7*	48	116
80	Zara × Maleta	P-19/10-3	*e1*	*E3*	*e4*	*e7*	*e1 E3 e4 e7*	49	116
81	Zara × Maleta	P-19/3-4	*E1*	*E3*	*E4*	*E7*	*E1 E3 E4 E7*	49	**135**
82	Zara × Soer 3	P-18/8-9	*E1*	*E3*	*E4*	*E7*	*E1 E3 E4 E7*	49	**136**
83	Zara × Ustya	P-20/2-1	*E1*	*E3*	*E4*	*E7*	*E1 E3 E4 E7*	49	**140**
84	Birlik KV × Pamyat YGK	P-10/2-9	*e1*	*E3*	*E4*	*e7*	*e1 E3 E4 e7*	50	**132**
85	Birlik KV × Pripyat	P-2/8-6	*e1*	*E3*	*E4*	*e7*	*e1 E3 E4 e7*	50	**134**
86	Zara × Maleta	P-19/10-8	*E1*	*E3*	*e4*	*E7*	*E1 E3 e4 E7*	50	106
87	Zara × Ustya	P-20/1-1	*e1*	*E3*	*E4*	*e7*	*e1 E3 E4 e7*	50	118
88	Zara × Jhony	P-13/1-6	*E1*	*E3*	*e4*	*E7*	*E1 E3 e4 E7*	51	**134**
89	Zara × Maleta	P-19/5-8	*e1*	*E3*	*e4*	*e7*	*e1 E3 e4 e7*	51	118
90	Zara × Maleta	P-19/1-3	*E1*	*E3*	*E4*	*E7*	*E1 E3 E4 E7*	51	**134**
91	Zara × Maleta	P-19/10-5	*E1*	*E3*	*E4*	*E7*	*E1 E3 E4 E7*	51	**135**
92	Zara × Bara	P-17/3-4	*E1*	*E3*	*E4*	*E7*	*E1 E3 E4 E7*	52	**136**
93	Zara × Maleta	P-19/10-2	*e1*	*E3*	*e4*	*e7*	*e1 E3 e4 e7*	52	100
94	Zara × Soer 3	P-18/3-2	*E1*	*E3*	*E4*	*E7*	*E1 E3 E4 E7*	52	**136**
95	Birlik KV × Gignon 5	P-3/1-5	*E1*	*E3*	*E4*	*E7*	*E1 E3 E4 E7*	53	**130**
96	Zara × Maleta	P-19/1-8	*E1*	*E3*	*E4*	*E7*	*E1 E3 E4 E7*	53	**136**
97	Zara × Ustya	P-20/5-1	*E1*	*E3*	*E4*	*E7*	*E1 E3 E4 E7*	53	**140**
98	Zara × Ustya	P-20/2-6	*E1*	*E3*	*e4*	*E7*	*E1 E3 e4 E7*	53	**136**
99	Zara × Soer 3	P-18/8-1	*E1*	*E3*	*E4*	*E7*	*E1 E3 E4 E7*	54	**137**
100	Birlik KV × Toury	P-6/4-5	*e1*	*E3*	*E4*	*e7*	*e1 E3 E4 e7*	55	**140**
101	Zara × Jhony	P-13/1-5	*E1*	*E3*	*e4*	*E7*	*E1 E3 e4 E7*	55	**139**
102	Zara × Maleta	O-47/11	*e1*	*E3*	*e4*	*e7*	*e1 E3 e4 e7*	57	108
103	Zara × Soer 3	P-18/5-6	*E1*	*E3*	*E4*	*E7*	*E1 E3 E4 E7*	58	**137**

Supplementary material S5. Growth and yield traits in plants of 20 selected breeding lines grown in field trials in northern Kazakhstan, Kostanay, 2022.

N	Hybrid	Breeding line ID	Genotype	Seed weight per plant (g)	1000 seed weight (g)	Time to maturity (days)
1	Birlik KV × Rana	P-1/11-5	*e1 e3 E4 e7*	12,3±1,6	135±4,6	94
2	Birlik KV × Rana	P-1/2-3	*e1 e3 E4 e7*	12,5±1,3	142±5,9	94
3	Birlik KV × Rana	P-1/10-2	*e1 e3 E4 e7*	12,1±1,5	138±2,8	94
4	Birlik KV × Rana	P-1/2-2	*e1 e3 E4 e7*	13,2±2,1	130±1,7	94
5	Birlik KV × Rana	P-1/10-5	*e1 e3 E4 e7*	16,1±1,6	136±3,1	95
6	Birlik KV × Rana	P-1/8-4	*e1 e3 E4 e7*	13,2±1,2	124±2,5	96
7	Birlik KV × Rana	P-1/10-3	*e1 e3 E4 e7*	13,5±1,5	129±1,9	96
8	Birlik KV × Rana	P-1/6-4	*E1 e3 E4 E7*	17,1±1,5	148±2,3	101
9	Birlik KV × Rana	P-1/8-3	*e1 e3 E4 e7*	15,2±1,6	145±2,7	101
10	Birlik KV × Rana	P-1/3-1	*e1 e3 E4 e7*	14,2±1,8	146±3,9	102
11	Birlik KV × Rana	P-1/9-5	*E1 e3 E4 E7*	17,8±2,4	138±2,5	102
12	Birlik KV × Rana	P-1/11-1	*e1 e3 E4 e7*	15,6±1,6	126±2,9	102
13	Birlik KV × Rana	P-1/7-4	*E1 E3 E4 E7*	17,9±2,3	129±3,6	102
14	Birlik KV × Rana	P-1/3-3	*e1 e3 E4 e7*	15,6±1,4	138±3,4	102
15	Birlik KV × Pripyat	P-2/5-4	*e1 E3 e4 e7*	18,2±2,2	156±5,2	92
16	Zara × Maleta	P-19/10-2	*e1 E3 e4 e7*	15,3±1,3	139±2,8	100
17	Birlik KV × Toury	P-6/4-1	*e1 E3 E4 e7*	15,6±2,4	129±2,5	94
18	Birlik KV × Soer 345	P-9/3-6	*E1 E3 E4 E7*	18,6±1,3	145±7,5	93
19	Birlik KV × Gignon 5	P-3/3-1	*e1 E3 E4 e7*	16,8±1,9	149±2,6	102
20	Birlik KV × Gignon 5	P-3/2-4	*e1 E3 E4 e7*	15,9±2,5	147±4,3	102
